# Divergent CD4^+^ T-cell profiles are associated with anti-HLA alloimmunization status in platelet-transfused AML patients

**DOI:** 10.3389/fimmu.2023.1165973

**Published:** 2023-08-28

**Authors:** Mehdi Khelfa, Mathieu Leclerc, Stéphane Kerbrat, Yakout Nait Sidenas Boudjemai, Médine Benchouaia, Déborah Neyrinck-Leglantier, Léonie Cagnet, Lylia Berradhia, Marie Tamagne, Laure Croisille, France Pirenne, Sébastien Maury, Benoît Vingert

**Affiliations:** ^1^ Établissement Français du Sang, Île-de-France, France; ^2^ Univ Paris Est Creteil, INSERM, IMRB, Équipe Pirenne, Créteil, France; ^3^ Laboratory of Excellence GR-Ex, Paris, France; ^4^ Assistance Publique - Hôpitaux de Paris, Hôpital Henri Mondor, Service d’Hématologie clinique, Créteil, France; ^5^ Univ Paris Est Creteil, INSERM, IMRB, Plateforme de Génomique, Créteil, France

**Keywords:** acute myeloid leukemia, alloimmunization, multi-omics, CD4+ T lymphocytes, platelet transfusion

## Abstract

**Introduction:**

Acute myeloid leukemia (AML) is one of the commonest hematologic disorders. Due to the high frequency of disease- or treatment-related thrombocytopenia, AML requires treatment with multiple platelet transfusions, which can trigger a humoral response directed against platelets. Some, but not all, AML patients develop an anti-HLA immune response after multiple transfusions. We therefore hypothesized that different immune activation profiles might be associated with anti-HLA alloimmunization status.

**Methods:**

We tested this hypothesis, by analyzing CD4+ T lymphocyte (TL) subsets and their immune control molecules in flow cytometry and single-cell multi-omics.

**Results:**

A comparison of immunological status between anti-HLA alloimmunized and non-alloimmunized AML patients identified differences in the phenotype and function of CD4+ TLs. CD4+ TLs from alloimmunized patients displayed features of immune activation, with higher levels of CD40 and OX40 than the cells of healthy donors. However, the most notable differences were observed in non-alloimmunized patients. These patients had lower levels of CD40 and OX40 than alloimmunized patients and higher levels of PD1. Moreover, the Treg compartment of non-alloimmunized patients was larger and more functional than that in alloimmunized patients. These results were supported by a multi-omics analysis of immune response molecules in conventional CD4+ TLs, Tfh circulating cells, and Tregs.

**Discussion:**

Our results thus reveal divergent CD4+ TL characteristics correlated with anti-HLA alloimmunization status in transfused AML patients. These differences, characterizing CD4+ TLs independently of any specific antigen, should be taken into account when considering the immune responses of patients to infections, vaccinations, or transplantations.

## Introduction

Platelet transfusions are an essential component of treatment to maintain homeostasis in patients with bleeding disorders. The development of platelet transfusion guidelines has facilitated chemotherapy management for patients with hematologic malignancies. More than a million new cases of hematologic malignancy are diagnosed annually, and the current management of these patients includes multiple prophylactic or therapeutic platelet transfusions.

However, some of these patients on long-term polytransfusion programs develop posttransfusion alloimmunization (1). The immune causes of this condition involve the production of alloantibodies against human platelet alloantigens, in most cases mismatched HLA class I molecules. Apart from occasionally causing a refractory state, which remains a rare condition, these antibodies can lead to a decrease in transfusion efficacy, resulting in an increase in the number of transfusions (2).

However, up to 80% of patients never develop class I HLA alloimmunization despite undergoing a large number of HLA-mismatched platelet transfusions on a regular basis (3, 4). The exact determinants of anti-platelet immune responses are not well understood. Leukoreduction reduces alloimmunization rates (5), but recipient CD4^+^ T lymphocytes (TLs) play a key role (6).

In a first study with patients with hematologic malignancies, we showed that these anti-HLA specific CD4^+^ T responses are present in alloimmunized patients (7). Regardless of the antigen studied, these CD4^+^ T-cell responses had similar, rather monofunctional profiles different from more conventional responses (*e.g.* those directed against pathogens). This observation suggested that these characteristics were related to the tumor environment, the transfusions themselves, or the characteristics of alloimmunized patients.

Interestingly, the characteristics of TLs are also informative in red blood cell (RBC) alloimmunization, and patients with hematologic malignancies also receive transfusions of RBCs (8). The secretion profiles of CD4^+^ TLs are also unconventional in this context, identifying several determinants as potentially associated with alloimmune or non-alloimmune status (9). Circulating T follicular helper cells (cTfh), Th17 and T regulatory cells (Tregs) can be used to differentiate between RBC-alloimmunized and non-alloimmunized patients (10–16).

All these transfusion alloimmunization data suggest that there may be differences in CD4^+^ T-cell immune activation state between alloimmunized and non-alloimmunized patients after platelet transfusion. Immunogenicity and alloimmunization are multifactorial, and will, therefore, always be difficult to predict. However, the identification of immunological features differing between alloimmunized and non-alloimmunized patients would make it possible to characterize the immune system of patients before immunotherapies. For the purposes of this study, we used acute myeloid leukemia (AML) as a posttransfusion alloimmunization model. AML is one of the most frequent forms of leukemia and its diagnosis and treatment guidelines are highly consistent (17–19). AML patients undergoing chemotherapy frequently suffer from thrombocytopenia. AML management therefore includes prophylactic or therapeutic platelet component transfusions (20). The prevalence of platelet-specific antibodies in AML does not differ significantly from that in other hematologic malignancies, at 20% to 30% of patients undergoing transfusion with protocols similar to those used at our center (21). RBC-alloimmunization rates are low in these patients, at up to 7% of patients (this value is also not significantly different from that for other hematologic malignancies) (22).

We studied the CD4^+^ TLs in the whole blood of two groups of AML patients: non-alloimmunized and alloimmunized, during transfusion and new alloantibody detection. We used polychromatic flow cytometry to investigate the molecules in CD4^+^ TLs involved in the immunomodulation of adaptive responses. The antibody panels used included antibodies against receptors from the CD28 superfamily, receptors from the TNF receptor superfamily, immune checkpoints, and Toll-like receptors (TLRs), as described in other alloimmunization models (9, 10, 12–16). We investigated the distribution and function of subpopulations of CD4^+^ TLs,including Th17, cTfh, and Tregs, all of which could potentially be involved in class I HLA alloimmunization (9, 10, 16).

Analyses based on single-cell RNAseq or multi-omics have already been performed for AML patients (23), but no previous study has directly addressed the relationship between alloimmunization and the CD4^+^ TL subpopulations present in these patients. We, therefore, performed a multi-omics analysis combining an immune gene response panel and antibodies targeting cell-surface proteins, to study the CD4^+^ TL subpopulations with a high degree of precision, based on their protein profiles.

## Materials and methods

### Patient groups

Two groups of polytransfused adult AML patients were included in this study: alloimmunized (*n*=20) and non-alloimmunized (*n*=22) patients. Another group of adult AML patients sampled before platelet transfusion (*n*=9) was also included in this study. All patients were recruited at Henri Mondor Hospital.

These patients had undergone transfusions (without transplantation) for AML at the time of whole-blood sampling (**Supplemental Table 1, 2**). Alloimmunized patients displayed alloimmunization against at least one new class I HLA antigen at the time of sampling (**Supplemental Table 3**). None of the patients displayed alloimmunization against an RBC antigen or against HPA molecules. For alloimmunized and non-alloimmunized patients, the last platelet component transfusion was performed no more than six days before blood collection, a time window matching that for antigenic stimulation. No significant difference was found between patients in terms of platelet transfusion efficacy (**Supplemental Figure 1**).

The control group consisted of healthy blood donors (HDs; *n*=28) from the *Etablissement Français du Sang* (**Supplemental Table 1**).

None of the participating patients or HDs had had an infection (bacterial, viral, fungal, yeast) or had been vaccinated in the 30 days preceding inclusion, and all gave written informed consent. None of the alloimmunized patients was receiving any specific treatment other than hypomethylating agents and chemotherapy at the onset of new anti-HLA antibody production. Subjects with a history of anti-HLA antibody production were excluded.

### Whole-blood phenotyping

Fresh whole blood was used for T-cell phenotyping by flow cytometry, without separation, as separation procedures can alter chemokine receptor expression (24). Briefly, cells were labeled with the Aqua LIVE/DEAD (Thermo Fisher Scientific, Waltham, MA) viability stain and the antibodies described in **Supplemental Table 4**. RBCs were lysed with Lysing Buffer (BD Biosciences, San Jose, CA). Cells were fixed and permeabilized with the Fix and Perm kit (Thermo Fisher Scientific) according to the manufacturer’s instructions. Intracellular markers were detected with the antibodies described in **Supplemental Table 4**.

### Treg suppression assay

Fresh whole blood was collected from patients and HDs. PBMCs were isolated by density gradient centrifugation, as previously described (16), and labeled with Aqua LIVE/DEAD (Thermo Fisher Scientific). They were also stained with CD4-R718, CD25-PE-Cy7 (BD Biosciences), and CD127-BV421 (BioLegend, San Diego, CA)antibodies. Tregs (CD4^+^CD25^hi^CD127^lo^), conventional T cells (Tconv, CD4^+^CD25^-^CD127^hi^) and autologous feeder cells (CD4^-^) were purified by flow cytometry, resulting in levels of Tconv, Treg and feeder cell purity > 98%.

Treg suppression assays were performed as previously described (16). Briefly, 96-well plates were coated with 0.5 µg/mL anti-CD3 antibody (UCHT1, Beckman Coulter, Brea, CA) for Tconv stimulation. Tconv cells were stained with CFSE (0.6 μM, Thermo Fisher Scientific) and cultured at a maximum density of 5000 cells per well, on the 96-well anti-CD3 antibody-coated plate. X-irradiated (30 Gy, Faxitron CP160, Hologic, Marlborough, MA) autologous feeder cells (10 times the number of Tconv cells) were added. Tregs were added to the culture at the following Treg-to-Tconv ratios: 1:1, 1:2, 1:4, 1:8 and 1:16. Cells were cocultured in supplemented RPMI 1640 medium (10% FBS, 100 IU/mL penicillin, 100 mg/mL streptomycin, 1 mM sodium pyruvate and 1X MEM) for four days at 37°C under an atmosphere containing 5% CO_2_. After the four days of culture, cells were labeled with Aqua LIVE/DEAD (Thermo Fisher Scientific) stain to facilitate the removal of non-viable cells from the analysis. They were then stained with the following antibodies: CD3-BUV737, CD4-R718, CD25-PE-Cy7 (BD Biosciences) and CD127-BV421 (BioLegend). Cell division was assessed by timed-acquisition flow cytometry for CFSE^lo^ Tconv.

### Single-cell multi-omics assay

Fresh whole blood was collected from patients and HDs. PBMCs were isolated by density gradient centrifugation. Leukocytes were labeled with Aqua LIVE/DEAD (Thermo Fisher Scientific) stain for the removal of non-viable cells, and with the following antibodies: CD45RA-APC-H7, CD3-BV650 and CD4-R718 (BD Biosciences). CD45RA^-^CD3^+^CD4^+^ TLs were isolated from PBMCs by flow cytometry sorting. Targeted scRNA-seq analysis was performed with the BD Rhapsody Single-Cell Analysis System (BD Biosciences), according to the manufacturer’s instructions. Each sample was stained with 20 BD AbSeq antibodies (**Supplemental Table 5**). The BD Human Single-Cell Multiplexing Kit was used to multiplex up to seven samples per Rhapsody cartridge. For library construction, the samples were pooled before cartridge loading. The BD Rhapsody Immune Response Targeted Panel for Humans was used to assess mRNA levels for 399 genes (#633750).

### Statistical analysis

All analyses were performed with Prism 6.07 software (GraphPad, La Jolla, CA). The significance of differences was determined in Mann-Whitney and *post hoc* tests. All significant differences between groups (*P*<0.05) are reported on data plots. Non-significant differences between groups are not reported on data plots, other than for particular values that are discussed. For the data presented in tables, the significance of differences was determined in Mann-Whitney, one-way ANOVA, chi-squared, and Fisher’s exact tests.

For single-cell RNAseq, analyses were performed with SeqGeq software, and differentially expressed genes were identified in Kruskal-Wallis tests. Only genes with a *Q*<0.05 were considered differentially expressed.

## Results

### CD4^+^ TL subpopulations and alloimmunization status

**Figure 1:**
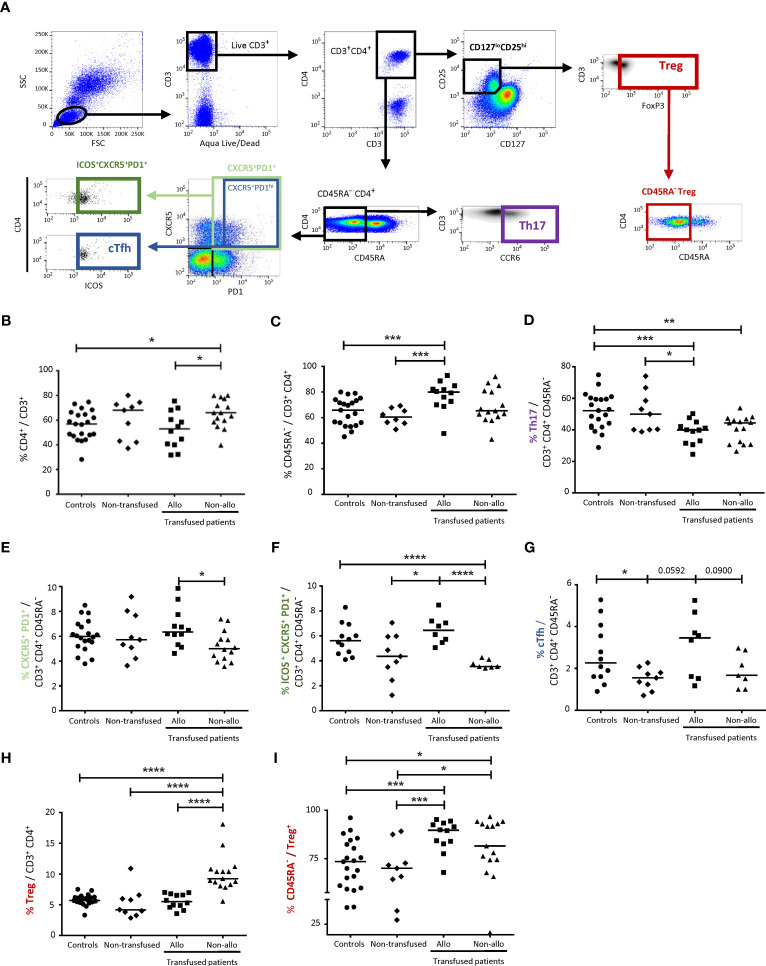
Comparison of CD4^+^ TL subpopulations between alloimmunized and non-alloimmunized patients. **(A)** Gating strategy for flow cytometry analysis. Lymphocytes were gated on size (forward scatter, FSC) and granularity (side scatter, SSC). Dead cells were excluded with Aqua LIVE/DEAD staining. CD4^+^ T lymphocytes (CD4^+^ TLs) were identified with the CD3^+^CD4^+^ gate. Activated Tconvs were identified with the CD45RA^-^ gate. T helper 17 cell (Th17) populations were identified with the CD3^+^CD4^+^CD45RA^-^CCR6^+^ gate. Circulating T follicular helper cell (cTfh) populations were identified with the CD3^+^CD4^+^CD45RA^-^CXCR5^+^PD1^hi^ICOS^+^ gate. T regulatory cell (Treg) populations were identified with the CD3^+^CD4^+^CD127^lo^CD25^hi^FoxP3^+^ gate and activated Tregs were identified with the CD45RA^-^ Treg gate. **(B)** Comparison of CD4^+^ TL percentages, **(C)** activated CD4^+^ TL percentages, **(D)** Th17 percentages, **(E)** CXCR5^+^PD1^+^ activated CD4^+^ TL percentages, **(F)** CXCR5^+^PD1^+^ ICOS^+^ activated CD4^+^ TL percentages, **(G)** cTfh percentages, **(H)** Treg percentages and **(I)** activated Treg percentages between alloimmunized (*n*=12, ▪, 12 experiments, with 1 donor per experiment; *n*=8 for CXCR5^+^PD1^+^ ICOS^+^ activated CD4^+^ TL and cTfh percentages), and non-alloimmunized (*n*=15, ▴, 15 experiments, with 1 donor per experiment; *n*=7 for CXCR5^+^PD1^+^ ICOS^+^ activated CD4^+^ TL and cTfh percentages) patients transfused with platelets and non-transfused patients (*n*=9, ♦, 9 experiments, with 1 donor per experiment). HDs (*n*=21, ●, 21 experiments, with 1 donor per experiment; *n*=12 for CXCR5^+^PD1^+^ ICOS^+^ activated CD4^+^ TL and cTfh percentages) were used as a control group. Horizontal bars indicate the median values. The significance of differences (*P*<0.05 considered significant) was determined in Mann-Whitney and *post hoc* tests. *P<0.05; **P<0.01; ***P<0.005; ****P<0.001.

CD4^+^ TL subpopulations were evaluated in whole blood by flow cytometry. Total CD4^+^, activated T CD4^+^ (CD45RA^-^), Tregs (CD25^hi^CD127^lo^FoxP3^+^CD4^+^), Th17 cells (CCR6^+^CD45RA^-^CD4^+^), cells with a Tfh profile (ICOS^+^CXCR5^+^PD1^+^) and cTfh (ICOS^+^CXCR5^+^PD1^hi^) cells were distinguished (**Figure 1A**).

The percentage of CD4^+^ TLs among total CD3^+^ lymphocytes was significantly higher in non-alloimmunized than in alloimmunized patients and controls (65.4 ± 11.5%, vs. 52.4 ± 14.7% and 56.0 ± 11.8%, respectively, *P*<0.05) (**Figure 1B**). No significant difference was found between alloimmunized patients, non-transfused patients and controls (**Figure 1B**). The percentage of activated CD4^+^ TLs among total CD4^+^ TLs was significantly higher in alloimmunized and non-transfused patients than in controls (77.1 ± 11.8% and 60.7% ± 6.5% vs. 64.6 ± 10.7% respectively, *P*<0.005) (**Figure 1C**). No significant difference was found between the two groups of transfused patients (**Figure 1C**).

The percentage of Th17 cells among activated CD4^+^ TLs was lower in alloimmunized and non-alloimmunized patients than in controls and non-transfused patients (38.7 ± 7.6% and 40.6 ± 8.3% respectively, vs. 51.7 ± 11.4%, *P*<0.005 and *P*<0.01, respectively, and vs. 51.3 ± 12.9) (**Figure 1D**). No difference was found between the two groups of transfused patients (**Figure 1D**).

The percentage of CXCR5^+^PD1^+^CD4^+^ TLs among activated CD4^+^ TLs was significantly higher in alloimmunized than in non-alloimmunized patients (6.7 ± 1.5% vs. 5.2 ± 1.2%, *P*<0.05) (**Figure 1E**). When the ICOS marker was added to the analysis, such that the observed cells were CD4^+^ TLs with a Tfh profile, the percentage of these cells among activated CD4^+^ TLs was significantly lower in non-alloimmunized than in alloimmunized patients and controls (3.7 ± 0.3% versus 6.5 ± 1.1% and 5.7 ± 1.3%, respectively, *P*<0.001) (**Figure 1F**). Considering only PD1^hi^ cells for the analysis of cTfh, we found that the percentage of these cells was lower in non-alloimmunized than in alloimmunized patients (1.9 ± 0.8% vs. 3.1 ± 1.5%) (**Figure 1G**).

The percentage of Tregs among CD4^+^ TLs was significantly higher in non-alloimmunized than in alloimmunized patients, non-transfused patients and controls (10.1 ± 3.0% vs. 5.6 ± 1.2%, 5.3 ± 2.5% and 5.8 ± 0.9%, respectively, *P*<0.001) (**Figure 1H**). The percentage of activated CD45RA^-^ Tregs among total Tregs was significantly higher in both alloimmunized and non-alloimmunized patients than in controls and non-transfused patients (86.0 ± 8.1% and 79.4 ± 20.2%, respectively vs. 71.1 ± 14.6% and 65.3 ± 20.3%, *P*<0.005 and *P*<0.05, respectively) (**Figure 1I**). No significant difference was found between the two groups of transfused patients, or between controls and non-transfused patients (**Figure 1I**).

### T regulatory cell function and alloimmunization status

**Figure 2:**
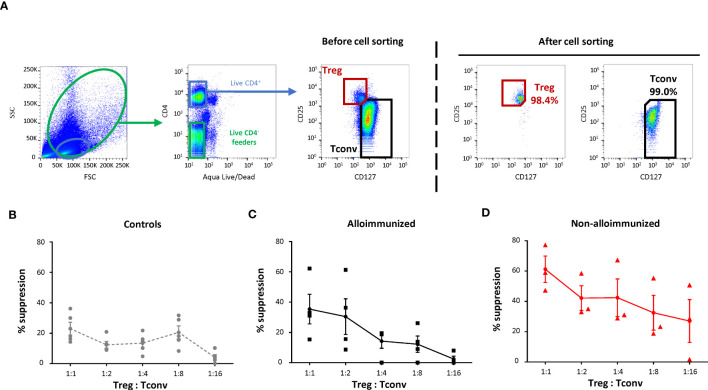
Comparison of the T-regulatory cell suppression function between alloimmunized and non-alloimmunized patients. **(A)** Gating strategy for flow cytometry cell sorting. Total PBMCs and lymphocytes were gated on size (forward scatter, FSC) and granularity (side scatter, SSC). Dead cells were excluded by Aqua LIVE/DEAD staining. Autologous feeder cells were sorted with the CD4^-^ live gate. Treg cells were sorted with the CD4^+^CD25^hi^CD127^lo^ gate. Conventional T cells (Tconv) were sorted with the CD4^+^CD25^lo^CD127^hi^ gate. **(B)** Proliferation of purified Tconv, cocultured with autologous purified Tregs at various Treg : Tconv ratios, supplemented with autologous purified feeder cells after stimulation in plates coated with anti-CD3 antibody. The percent suppression was calculated as follows: 100 - (% CFSE^lo^ Tconv proliferation/% CFSE^lo^ Tconv proliferation when cultured without Tregs) x 100. The mean percent suppression values (± SEM) were calculated for **(B)** controls (●, *n*=5), **(C)** alloimmunized patients (▪, *n*=4) and **(D)** non-alloimmunized patients (▴, *n*=3). Points indicate the percent suppression values for each experiment, with vertical bars indicating the SEM. The significance of differences (*P*<0.05 considered significant) was determined in Mann-Whitney tests.

We compared the *in vitro* suppressive activity of Tregs between alloimmunized and non-alloimmunized patients. We also compared this activity between patient groups and a control group. Treg, Tconv and autologous feeder cells were obtained by flow cytometry sorting (**Figure 2A**).

The percent Treg suppression in alloimmunized patients was not significantly different from that in controls (**Figures 2B, C**). Treg suppression was significantly higher in non-alloimmunized patients than in controls (P<0.01, Mann-Whitney) (**Figures 2B–D**). Treg suppression was also significantly higher in non-alloimmunized than in alloimmunized patients (P<0.05, Mann-Whitney) (**Figures 2C, D**).

### Immunomodulatory molecules and alloimmunization status

CD4^+^ T-cell alloimmunization determinants were compared between patient groups by flow cytometry. For CTLA4, LAG3, TIM3, BTLA and TIGIT expression in activated CD4^+^ TLs, we found no significant difference between patient groups (**Supplemental Figure 2**).

**Figure 3:**
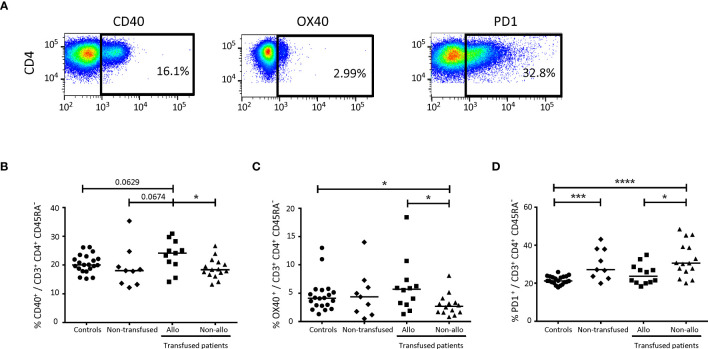
Comparison of immunomodulatory molecules on CD4^+^ TLs between alloimmunized and non-alloimmunized patients. **(A)** Gating strategy for flow cytometry analysis. Analyses were performed on activated CD4^+^ TLs, gated as shown in **Figure 1**. **(B)** Comparison of CD40^+^, **(C)** OX40^+^ **(D)** and PD1^+^ cell percentages in whole-blood activated CD4^+^ TLs between alloimmunized (*n*=11, ▪, 11 experiments, with 1 donor per experiment), and non-alloimmunized (*n*=14, ▴, 14 experiments, with 1 donor per experiment) patients transfused with platelets and non-transfused patients (*n*=9, ♦, 9 experiments, with 1 donor per experiment). HDs (*n*=20, ●, 20 experiments, with 1 donor per experiment) were used as a control group. Horizontal bars indicate the median values. The significance of differences (*P*<0.05 considered significant) was determined in Mann-Whitney and *post hoc* tests. *P<0.05; ***P<0.005; ****P<0.001.

Differences were found only for CD40, OX40 and PD1 (**Figure 3A**). The percentage of activated CD4^+^ TLs expressing CD40 was significantly higher in alloimmunized than in non-alloimmunized patients (23.4 ± 5.4% vs. 18.2 ± 2.9%, *P*<0.01). The percentage of activated CD4^+^ TLs expressing CD40 was also higher in alloimmunized than in non-transfused patients and controls (23.4 ± 5.4% vs. 19.2 ± 7.2% and 20.3 ± 3.3%, respectively) (**Figure 3B**).

The percentage of activated CD4^+^ TLs expressing OX40 was significantly lower in non-alloimmunized than in alloimmunized patients and controls (2.9 ± 1.9% vs. 6.5 ± 4.8% and 4.6 ± 2.9%, respectively, *P*<0.05) (**Figure 3C**). No difference was found between non-transfused patients and controls (**Figure 3C**).

The percentage of activated CD4^+^ TLs expressing PD1 was significantly higher in non-alloimmunized than in alloimmunized patients and controls (32.8 ± 9.2% vs. 24.6 ± 5.6% and 21.5 ± 2.1%, respectively, *P*<0.05 and *P*<0.001, respectively) (**Figure 3D**). The percentage of activated CD4^+^ TLs expressing PD1 was also significantly higher in non-transfused patients than in controls (30.1 ± 8.1% vs. 21.5 ± 2.1%, respectively, *P*<0.005). No difference was found between non-transfused patients and transfused patients (**Figure 3D**).

### Toll-like receptors and alloimmunization status

Flow cytometry was used to evaluate TLR expression. We found no significant differences between patient groups for TLR2, TLR9, and TLR10 expression on activated CD4^+^ TLs (**Supplemental Figure 3**).

**Figure 4:**
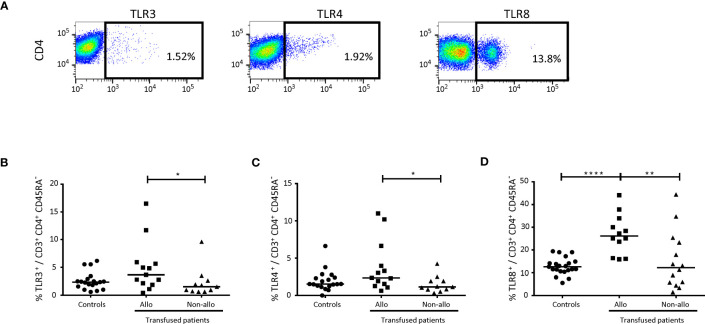
Comparison of Toll-like receptors on CD4^+^ TLs between alloimmunized and non-alloimmunized patients. **(A)** Gating strategy for flow cytometry analysis. Analyses were performed on activated CD4^+^ TLs, gated as shown in **Figure 1**. **(B)** Comparison of TLR3^+^ **(C)** and TLR4^+^ percentages in whole-blood activated CD4^+^ T lymphocytes between alloimmunized (*n*=13, ▪, 13 experiments, with 1 donor per experiment) and non-alloimmunized (*n*=11, ▴, 11 experiments, with 1 donor per experiment) patients. HDs (*n*=19, ●, 19 experiments, with 1 donor per experiment) were used as a control group. **(D)** Comparison of TLR8^+^ percentages in whole-blood activated CD4^+^ T lymphocytes between alloimmunized (*n*=12, ▪, 12 experiments, with 1 donor per experiment), and non-alloimmunized (*n*=14, ▴, 14 experiments, with 1 donor per experiment) patients. HDs (*n*=21, ●, 21 experiments, with 1 donor per experiment) were used as a control group. Horizontal bars indicate the median values. The significance of differences (*P*<0.05 considered significant) was evaluated in Mann-Whitney and *post hoc* tests. *P<0.05; **P<0.01; ****P<0.001.

Significant differences were detected only for TLR3, TLR4 and TLR8 (**Figure 4A**). The percentage of activated CD4^+^ TLs expressing TLR3 was significantly higher in alloimmunized than in non-alloimmunized patients (5.0 ± 4.5% vs. 2.3 ± 2.6%, *P*<0.05) (**Figure 4B**). The percentage of activated CD4^+^ TLs expressing TLR4 was also significantly higher in alloimmunized than in non-alloimmunized patients (3.8 ± 3.4% vs. 1.5 ± 1.1%, *P*<0.05) (**Figure 4C**). The percentage of activated CD4^+^ TLs expressing TLR8 was significantly higher in alloimmunized than in non-alloimmunized patients and controls (27.0 ± 8.7% vs. 15.1 ± 12.3% and 12.8 ± 3.7%, respectively, *P*<0.01 and *P*<0.001, respectively) (**Figure 4D**).

### CD4^+^ TL multi-omics and alloimmunization status

**Figure 5:**
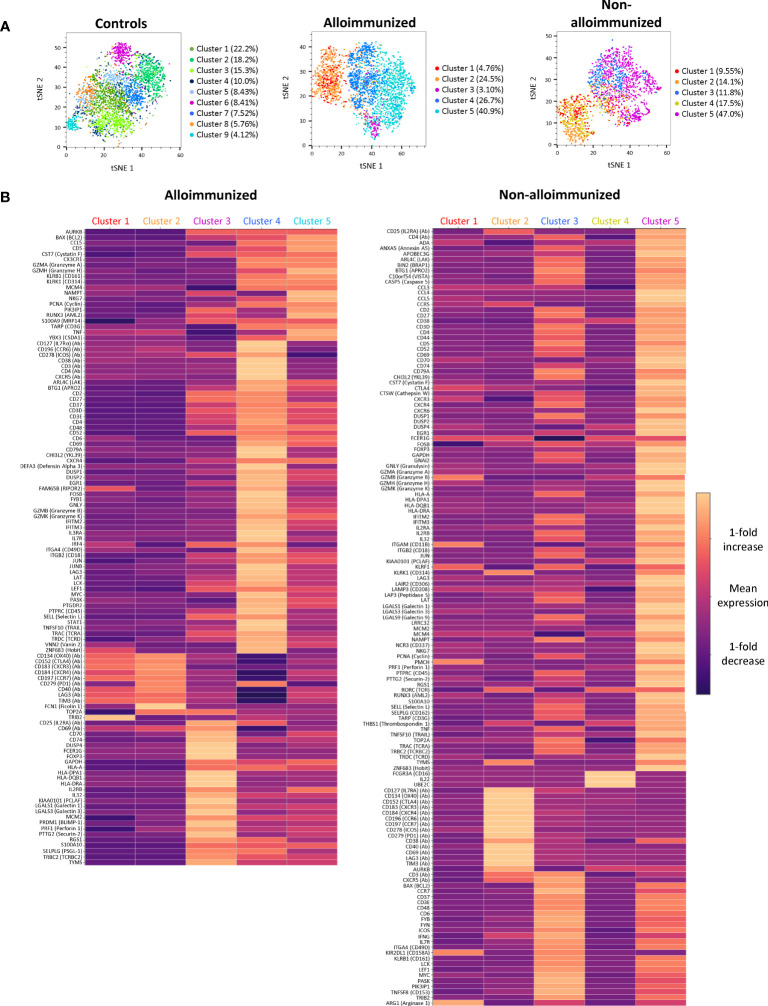
Clustering and comparison of CD45RA^-^ CD4^+^ TL mRNA and protein levels between alloimmunized and non-alloimmunized patients. Single-cell RNAseq and protein analysis were performed for *n*=7 individuals per group (controls, alloimmunized patients, and non-alloimmunized patients). **(A)** tSNE visualization of cells, each color representing a different phenotypic cluster. **(B)** Heatmap of the differentially expressed genes for each of the clusters highlighted in alloimmunized patients and non-alloimmunized patients. The color scale indicates the mean fold-change in expression.

We used single-cell RNAseq combined with protein analysis to confirm the differences for all CD4^+^ TL between the immune profiles of AML patient groups. A differential distribution of phenotypic clusters was observed between groups of patients (**Figures 5A, B**).

**Figure 6:**
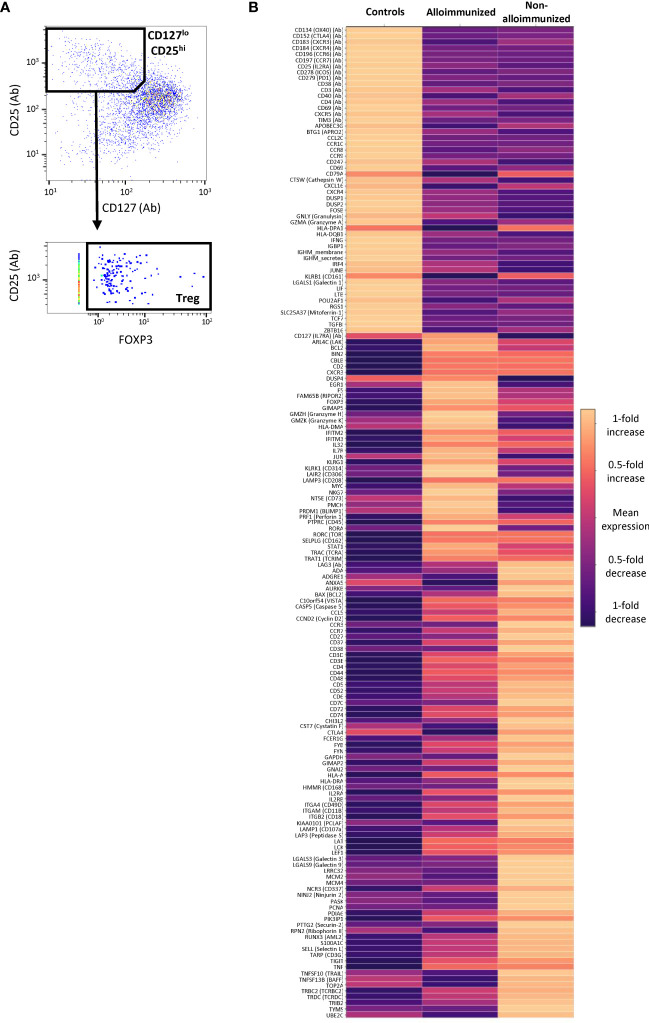
Comparison of CD45RA^-^ CD4^+^ Treg mRNA and protein levels between alloimmunized and non-alloimmunized patients. Single-cell RNAseq and protein analysis were performed for *n*=7 individuals per group (controls, alloimmunized patients, and non-alloimmunized patients). **(A)** Gating strategy for differential expression analysis. Treg cells were identified as CD127^lo^CD25^hi^FoxP3^+^ cells. **(B)** Heatmap of the genes differentially expressed in Treg cells for controls, alloimmunized patients and non-alloimmunized patients. The color scale indicates the mean fold-change in expression.

Using multi-omics analysis, we gated CD127 and CD25 protein expression with *FOXP3* gene expression to study Tregs (**Figure 6A**). Tregs from non-alloimmunized patients, alloimmunized patients, and controls had different profiles (**Figure 6B**).

Tregs from non-alloimmunized patients had higher expression levels for a panel of markers, including LAG3 protein and the *KLRB1*, *ADGRE1*, *ANXA5*, *CCR3*, *CD27*, *CD38*, *CST7*, *CTLA4*, *GAPDH*, *GNAI2*, *HMMR*, *IL2RB*, *PCLAF*, *LGALS3*, *LGALS9*, *LRRC32*, *MCM2*, *MCM4*, *PASK*, *PCNA*, *PTTG2*, *RPN2*, *TOP2A* and *UBE2C* genes, than Tregs from alloimmunized patients and controls (**Figure 6B**).

Tregs from alloimmunized patients displayed higher levels of expression for the CD127 protein, and the *PRDM1*, *DUSP4*, *EGR1*, *RIPOR2*, *MYC*, and *NT5E* genes than Tregs from non-alloimmunized patients and controls (**Figure 6B**).

## Discussion

Few studies have focused on posttransfusion alloimmunization in patients with hematologic malignancies, even though this immunization provides useful information about the characteristics of the patient’s immune system.

We found that non-alloimmunized patients had a slightly higher percentage of CD4^+^ TLs. However, alloimmunized patients had higher percentages of CD4^+^ TLs differentiated towards an activated phenotype than non-alloimmunized patients. This finding may be explained by the differential expression of CD4^+^ TL subpopulations. We therefore studied all CD4^+^ TL subpopulations involved in other types of alloimmunization: Th17 cells, Tfh and Tregs (9, 12, 16).

The percentage of CD4^+^ Th17 cells among activated CD4^+^ TLs was lower in the two groups of patients than in controls. Unexpectedly, Th17 profile did not differentiate between patients as a function of their alloimmunized/non-alloimmunized status. RBC alloimmunization leads to CD4^+^ TL differentiation, resulting in a Th17 profile in 56% of alloimmunized patients, whereas treated AML patients had lower frequencies of Th17 cells (7, 9, 25, 26).

Tfh-like subsets have been shown to be present at high levels in RBC-non-alloimmunized patients; we therefore investigated these cells in our AML patients (9, 15). We found that non-alloimmunized patients had lower percentages of CXCR5^+^PD1^+^ CD4^+^ TLs among activated CD4^+^ TLs, confirming the weak functional profile of CD4^+^ T responses in this group of patients. Even stronger support for this hypothesis was obtained when ICOS was added to the analysis. Non-alloimmunized patients had even lower levels of ICOS^+^ Tfh-like cells. These data also suggest that there is less differentiation towards the Tfh phenotype in non-alloimmunized patients. ICOS is a regulator of many key events in Tfh differentiation, indirectly controlling the function of these cells (27–32). Finally, cTfh cells, which are characterized by high levels of PD1 expression (33, 34), were also present at a lower frequency in non-alloimmunized patients.

We hypothesized that Treg subset levels might reflect the percentage of Tfh cells in non-alloimmunized patients. Previous studies showed that large numbers of potent Tregs are present in AML patients regardless of their alloimmunization status (35, 36). Tregs are involved in downregulating the antitumoral response and are associated with a poor prognosis (37–41), but these studies did not consider possible differences in the levels of these cells between subgroups of transfused patients.

Treg levels were, indeed, markedly higher in non-alloimmunized patients than in alloimmunized patients and controls. We investigated the activation of Tregs. Activated Tregs have greater suppressive and proliferative capabilities than their naïve counterparts (42). The percentages of activated Tregs were similar in the two patient groups. Activated Treg levels increase with age (43), potentially accounting for the high percentage of activated Tregs in both patient groups, given the median age of AML patients.

Overall, these data suggest a more immunosuppressive profile for non-alloimmunized patients, with no defects of Treg differentiation in alloimmunized patients. We investigated whether Tregs played an inhibitory role in non-alloimmunized patients, by assessing the capacity of these cells to suppress the proliferation of conventional T cells *in vitro* relative to that of Tregs from alloimmunized patients. We found that Tregs from non-alloimmunized were much more suppressive than those from alloimmunized patients.

We show here that it is possible to distinguish two groups of AML patients with different immune statuses. This distinction was based on the absence of alloimmunization and a correlation with the strong functional suppressive activity of Tregs. The multi-omics analysis on activated CD4^+^ TLs confirmed that these two types of patients had specific characteristics. It remains difficult to understand the precise immune functions of these clusters, because these patients have no functional defect of CD4^+^ TLs. Furthermore, comparisons with other multi-omics analyses are also difficult to evaluate, because these studies did not consider alloimmunized or non-alloimmunized status and did not examine the same characteristics as our study (23). We focused on Tfh and Treg cells, using the protein profiles defining these subpopulations to facilitate single-cell RNASeq analysis.

Tregs from both patient groups were functional and displayed no defects. However, multi-omics analysis highlighted two different activation profiles, both of which differed from the HD Treg profile. Tregs from alloimmunized patients had a more activated phenotype, with the generation of a larger number of mRNAs than Tregs from HDs, including the transcripts of *PTPRC* and *TIGIT* (44, 45), but without functional benefit. Tregs from non-alloimmunized patients also had a more activated phenotype than those from HDs. Moreover, they differed from the Tregs of alloimmunized patients in that they expressed other immune regulation-associated mRNAs involved in improving Treg maintenance and function: *CTLA4* and *LAG3* (46, 47). Other mRNAs upregulated included the chemokine receptor *CCR3*, the ligand of which, CCL11, may increase the proportion of Tregs (48). Survival- and proliferation-associated mRNAs were also upregulated in the Tregs of non-alloimmunized patients: *CD70*, *CD27*, *CD38* and *IL2RB* (48, 49). In addition, the Tregs of non-alloimmunized patients were shown to express several mRNAs involved in mitotic processes, such as those encoding topoisomerase TOP2A, which is essential for T-cell function (50), and metabolic mediators, such as PASK (51).

Highly suppressive Treg activity in non-alloimmunized patients also has consequences for Tfh cells. Multi-omics again revealed a different profile between alloimmunized, non-alloimmunized patients and controls. Tfhs from alloimmunized patients had higher levels of mRNAs associated with activation, such as *CD27*, indicating a highly activated phenotype (**Supplemental Figure 4**). Surprisingly, Tfhs from non-alloimmunized patients also had an activated phenotype, but with key differences relative to the Tfhs of alloimmunized patients. They had larger amounts of surface proteins, including immunoregulatory molecules, such as CTLA4 and LAG3 (**Supplemental Figure 4**). We hypothesize that this unusual profile may be a direct consequence of Treg inhibition. However, one of the limitations of this study is that the cells observed were circulating Tfhs. Access to secondary lymphoid organs would be required for confirmation of this finding without extrapolating the impact of Treg inhibition of the Tfh subset.

In addition to the impact of Tregs on Tfhs, this activity has broader consequences for the activation of conventional CD4^+^ TLs. We assessed this impact by studying immunomodulation markers and TLR expression in conventional CD4^+^ TLs. We hypothesized that several immune activation markers might distinguish between different immune groups of AML patients.

The first marker we identified was CD40 in alloimmunized patients. This result was expected, as CD40 is a prosurvival molecule with a costimulatory function for activated CD4^+^ TLs (52, 53). High levels of CD40 are suggestive of an active phenotype favoring immune responses, such as alloimmunization (54).

Like CD40, OX40 is a marker of anti-RBC responses and was characteristically expressed in alloimmunized patients in our study. This finding is particularly interesting, because OX40 plays a role in expansion and survival, but also in the cytokine production of conventional CD4^+^ TLs (55), by inhibiting Treg function, in particular (56).

Independently of CD40 and OX40, we found that PD1 was strongly upregulated in the CD4^+^ TLs of non-alloimmunized patients. This high level of PD1 expression suggests a possible limitation of CD4^+^ TL activation in non-alloimmunized patients, in addition to an exhausted phenotype characterized by the expression of this marker (57, 58). The PD1/PDL1 axis is a widely described immune checkpoint involved in limiting the immune response of CD4^+^ TLs (59, 60). Regardless of the transfusion context, PD1 is upregulated in the CD4^+^ TLs of AML patients, but with considerable heterogeneity between patients (61). This heterogeneity may reflect differences between alloimmunized and non-alloimmunized patients. We also found that PD1 was upregulated in the CD4^+^ TLs of non-transfused patients relative to controls, but with no significant differences relative to the two groups of transfused patients. These findings again highlight the discrepancy between the immune states of AML patients, as these non-transfused patients may or may not develop an anti-platelet response after transfusion.

The adaptive response is strongly linked to TLR stimulation on antigen-presenting cells, CD4^+^ TLs and even Tregs (9, 13, 62–64). By analogy with anti-RBC responses, we hypothesized that the differences in activated CD4^+^ TL levels between alloimmunized and non-alloimmunized patients could be explained by the stimulation of innate immunity via TLRs.

We found that the TLR2, TLR9 and TLR10 levels of CD4^+^ TLs did not differ significantly between groups of patients, but that TLR3, TLR4 and TLR8 levels were higher in alloimmunized patients. TLR3 and TLR4 ligands may induce the activation of CD4^+^ TLs in alloimmunized patients. Indeed, TLR stimulation induces the upregulation of activation markers, such as CD40 and OX40 (62, 63). This increase in TLR3/4 expression may be due to genetic factors, or to receptor transfer from extracellular vesicles (EVs) (65).

The high levels of TLR8 expression are particularly interesting. Indeed, TLR8 signaling can reverse the suppressive function of Tregs by selective inhibition (66, 67). The high frequencies of TLR8-positive activated CD4^+^ TLs in alloimmunized patients may make a major direct or indirect contribution to the alloimmune response. We hypothesize that this high level of TLR8 expression also results from the transfer of TLR proteins from platelet EVs present in the platelet concentrates (68, 69). Interestingly, no relevant differences in the levels of *TLR8* mRNA between cells were highlighted by our single-cell RNAseq experiments, supporting our hypothesis. We found no significant difference in TLR8 protein levels on activated CD4^+^ TLs between non-transfused patients and healthy donors (data not shown). Another hypothesis may account for the differences in TLR8 levels between groups. Indeed, several studies have shown that hypomethylating agents indirectly upregulate endogenous retroviral gene expression, mimicking an antiviral response by activating innate pattern recognition receptors, such as RNA-sensing TLRs (70–72). However, we found no correlation between the administration of hypomethylating agents and TLR8 overexpression (data not shown). A larger sample would be needed to test this hypothesis.

In summary, CD4^+^ TL determinants of platelet alloimmunization include OX40, CD40, PD1, TLR expression, and the proportions of TL subpopulations, including Tregs in particular (**Table 1**). These determinants may decrease the recipient’s ability to respond to platelet transfusion and may reflect the levels and suppressive function of their circulating Tregs. Even if no correlation was found between the patients’ characteristics and the immunological parameters studied (data not shown), these results represent a major breakthrough, as the management of AML patients has a strong immune component. However, as shown here, not all patients with AML have the same CD4^+^ T-cell response profile. These differences reflect a certain heterogeneity between patients and CD4^+^ T-cell response profile cannot, therefore, be considered a reliable marker for determining alloimmunization status. Nevertheless, single-cell multi-omics confirmed differences in immune activation profile between alloimmunized and non-alloimmunized patients. A prospective longitudinal multicenter clinical study on a larger sample of patients is now required, to make it possible to draw conclusions concerning the CD4^+^ T-cell determinants of platelet alloimmunization in AML patients. This study should follow patients from diagnosis, with the collection of data both before and after transfusion/alloimmunization. The EVs present in the bloodstream of these patients should also be investigated.

**Table 1:** Summary of findings.

		Alloimmunized	Non alloimmunized
CD4+ T-cell surface determinants	CD40	++	–
	OX40	+	–
	PD1	–	+
	TLR3	+	–
	TLR4	+	–
	TLR8	++	–
CD4+ T-cell subpopulations	Tfh	+++	–
	Treg	–	++++
Treg activity		No increase in activity relative to HDs	50% increase in activity relative to alloimmunized
Multi-omics	Treg	Lesser activation : CD127, LAG310, CTLA4lo, CD27lo, CD70lo, CD38lo	Higher activation : LAG3, CTLA4, CD27, CD70, CD38

Summary of the CD4^+^ T-cell determinants of platelet alloimmunization status identified in flow cytometry, functional analysis, and single-cell multi-omics analyses. +: *P*<0.05; ++: *P*<0.01; +++: *P*<0.005; ++++: *P*<0.001.

The identification of these immune profiles may help to improve the results of new immune treatments for AML (73–78), and to shed light on the major role potentially played by Tregs. As the changes observed concern global T-cell profiles (they are not antigen-specific), these findings should be taken into account when considering the patient’s responses to infections, vaccinations, and transplantations.

## Data availability statement

The datasets presented in this study can be found in online repositories. The names of the repository/repositories and accession number(s) can be found below: GSE230300 (GEO).

## Ethics statement

The studies involving human participants were reviewed and approved by CPP Ile de France. The patients/participants provided their written informed consent to participate in this study.

## Author contributions

BV was the principal investigator and takes primary responsibility for the paper. ML and SM recruited the patients. YB was the clinical research associate for this study. MK, LaC, MB and SK performed the laboratory work. MK, ML and BV analyzed the results. BV and SM coordinated the research. MK, MT, FP, LaC, SM and BV wrote the paper. DN-L, LeC and LB reviewed the paper. All authors contributed to the article and approved the submitted version.
