# Supplementary material for: Divergent CD4+ T-cell profiles are associated with anti-HLA alloimmunization status in platelet-transfused AML patients
Source: Front Immunol. 2023 Aug 28;14:1165973. doi: 10.3389/fimmu.2023.1165973 (PMC10493329; doi:10.3389/fimmu.2023.1165973)
Supplement: Supplementary file 1 [file DataSheet_1.docx]

**Divergent CD4^+^ T-cell profiles are associated with anti-HLA alloimmunization status in platelet-transfused AML patients**

Mehdi Khelfa^1-3^, Mathieu Leclerc^5^, Stéphane Kerbrat^4^, Yakout Nait Sidenas Boudjemai^5^, Médine Benchouaia,^4^ Déborah Neyrinck-Leglantier^1-3^, Léonie Cagnet^1-3^, Lylia Berradhia^1-3^, Marie Tamagne^1-3^, Laure Croisille^1^, France Pirenne^1-3^, Sébastien Maury,^5^ ^†^ and Benoît Vingert^1-3 †^

^1^ Établissement Français du Sang, Île-de-France, France.

^2^ Univ Paris Est Creteil, INSERM, IMRB, Équipe Pirenne, Créteil, 94000, France.

^3^ Laboratory of Excellence GR-Ex, Paris, 75739, France.

^4^ Univ Paris Est Creteil, INSERM, IMRB, Plateforme de Génomique, Créteil, 94000, France.

^5^ Assistance Publique - Hôpitaux de Paris, Hôpital Henri Mondor, Service d’Hématologie clinique, Créteil, 94000, France.

† These authors contributed equally to this work.

**Supplemental Data**

Supplemental Methods

Supplemental Method References

Supplemental Table 1: Characteristics of the study subjects.

Supplemental Table 2: Individual characteristics of alloimmunized and non-alloimmunized patients.

Supplemental Table 3: Transfusion characteristics of the study subjects.

Supplemental Table 4: Fluorescent antibodies for whole-blood phenotyping.

Supplemental Table 5: AbSeq antibodies for single-cell multi-omics analysis.

Supplemental Figure 1: Comparison of platelet transfusion efficacy between alloimmunized and non-alloimmunized patients.

Supplemental Figure 2: Comparison of immunomodulatory molecules on CD4^+^ TLs between alloimmunized and non-alloimmunized patients.

Supplemental Figure 3: Comparison of Toll-like receptors on CD4^+^ TLs between alloimmunized and non-alloimmunized patients.

Supplemental Figure 4: Comparison of CD45RA^-^ CD4^+^ cTfh mRNA and protein levels between alloimmunized and non-alloimmunized patients.

**Supplemental Methods**

**Monitoring of platelet survival**

Platelet counts for each alloimmunized and non-alloimmunized patient were collected before the last platelet transfusion (performed at the time of sampling) and 24 hours posttransfusion, together with the number of platelets in each of the transfused components (Supplemental Table 2). Corrected count increment (CCI) was used here to assess the response to platelet transfusion in each alloimmunized and non-alloimmunized patient. CCI was calculated as follows:^1^

$$CCI=\frac{\left( posttransfusion-pretransfusion platelet count \times{10}^{9} per L \right)\times body surface area (m^{2})}{number of platelets in component \times{10}^{11}}$$

Body surface area (BSA) for alloimmunized and non-alloimmunized patients was calculated with the Mosteller formula:^2^

$$BSA \left( m^{2} \right)= \sqrt{\frac{height \left( cm \right) \times weight (kg)}{3600}}$$

**HLA antibody detection**

The class I HLA antibodies present in serum samples were analyzed with a commercial bead-based multiplexing assay, in accordance with the manufacturer’s instructions (LABScreen Single Antigen Class I from ONE LAMBDA, Thermo Fisher Scientific, Waltham, MA) as previously described.^3^ The results were considered positive only if the values obtained were above the mean fluorescence intensity cutoff of 1500, as required by the French Committee for Accreditation in the framework of a graft protocol.

**Flow cytometers and fluorescence analysis**

For cell phenotyping, fluorescence was assessed on a LSRFortessa (BD Biosciences) flow cytometer; for cell isolation, cells were sorted on a FACSAria Fusion (BD Biosciences) flow cytometer. Flow cytometry data were analyzed with FlowJo software (v.10.7.1, FlowJo).

**Single-cell multi-omics analysis**

Sequencing was performed with an Illumina HiSeq X (Illumina, San Diego, CA) at the IMRB core facility. Fastq files were converted with the BD Rhapsody Analysis Pipeline (BD Biosciences) and analyzed with BD DataView software v.1.2.2 (BD Biosciences). Hierarchical clustering was performed in SeqGeq (v1.7, FlowJo, Ashland, OR) with the PhenoGraph cluster plugin, as described by Levine et al.^4^ Differential expression was visualized with heatmaps generated with the ViolinBox plugin. For clustering analyses, samples from the patients of a given group were concatenated. Final quality controls were performed on SeqGeq. Outlier events were removed based on the ratio of cell library size to the numbers of genes expressed. Highly dispersed genes were then gated separately on high-quality cells for each group of patients. The gated genesets were normalized and log-transformed for dimensionality reduction by principal component analysis (PCA) and t-distributed stochastic neighbor embedding (tSNE).

**Supplemental Method References**

1. Davis KB, Slichter SJ, Corash L. Corrected count increment and percent platelet recovery as measures of posttransfusion platelet response: problems and a solution. Transfusion (Paris). 1999;39(6):586-592.

2. Mosteller RD. Simplified calculation of body-surface area. N Engl J Med. 1987;317(17):1098.

3. Coombs J, Ben Hassen L, Leclerc M, et al. Dominant immune response to HLA-B57/B58 molecules after platelet transfusion. *Transfusion (Paris)*. 2020;60(12):2807-2814.

4. Levine JH, Simonds EF, Bendall SC, et al. Data-driven phenotypic dissection of AML reveals progenitor-like cells that correlate with prognosis. *Cell*. 2015;162(1):184-197.

**Supplemental Tables**

**Supplemental Table 1:** **Characteristics of the study subjects.**

**Supplemental Table 2:** **Individual characteristics of alloimmunized and non-alloimmunized patients.**

**Supplemental Table 3: Transfusion characteristics of the study subjects.**

**Supplemental Table 4: Fluorescent antibodies for whole-blood phenotyping.**

**Supplemental Table 5: AbSeq antibodies for single-cell multi-omics analysis.**

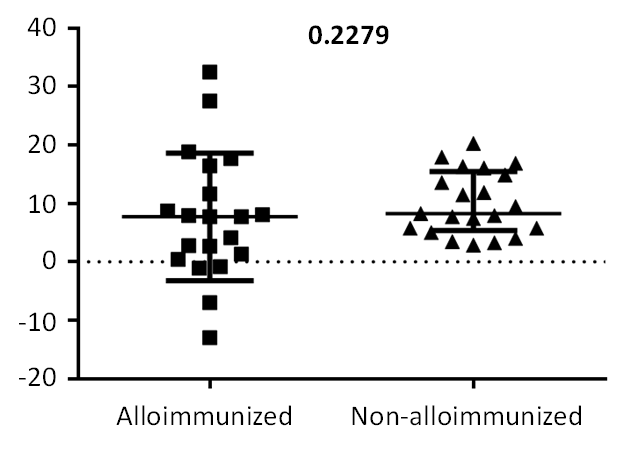
**Supplemental Figures**

CCI

**Supplemental Figure 1:** **Comparison of platelet transfusion efficacy between alloimmunized and non-alloimmunized patients.** Corrected count increment (CCI) was used to assess platelet transfusion efficacy in alloimmunized (*n*=20, ■) and non-alloimmunized (*n*=21, ▲) patients according to platelet count, body surface area and number of transfused platelets (Supplemental Table 2). CCI was calculated as described in the supplemental methods. Horizontal bars indicate the mean values ± SD. The significance of differences was evaluated in Mann-Whitney tests.

**Supplemental Figure 2: Comparison of immunomodulatory molecules on CD4^+^ TLs between alloimmunized and non-alloimmunized patients.** (A) Gating strategy for flow cytometry analysis. Analyses were performed on activated CD4^+^ TLs, gated as shown in Figure 1. (B) Comparison of CTLA4^+^, (C) LAG3^+^, (D) TIM3^+^, (E) BTLA^+^ and (F) TIGIT^+^ cell percentages for activated CD4^+^ TLs between alloimmunized patients (*n*=13, ■, 13 experiments, with 1 donor per experiment) and non-alloimmunized patients (*n*=11, ▲, 11 experiments, with 1 donor per experiment) transfused with platelets. HDs (*n*=19, ●, 19 experiments, with 1 donor per experiment) were used as a control group. Horizontal bars indicate the median values. The significance of differences (*P*<0.05 considered significant) was evaluated in Mann-Whitney and post hoc tests. **P*<0.05

**Supplemental Figure 3: Comparison of Toll-like receptors on CD4^+^ TLs between alloimmunized and non-alloimmunized patients.** (A) Gating strategy for flow cytometry analysis. Analyses were performed on activated CD4^+^ TLs, gated as shown in Figure 1. (B) Comparison of TLR2^+^, (C) TLR9^+^ and (D) TLR10^+^ cell percentages in activated CD4^+^ TLs between alloimmunized patients (*n*=13, ■, 13 experiments, with 1 donor per experiment) and non-alloimmunized patients (*n*=11, ▲, 11 experiments, with 1 donor per experiment) transfused with platelets. HDs (*n*=19, ●, 19 experiments, with 1 donor per experiment) were used as a control group. Horizontal bars indicate the median values.

**Supplemental Figure 4: Comparison of CD45RA^-^ CD4^+^ cTfh mRNA and protein levels between alloimmunized and non-alloimmunized patients.** Single-cell RNAseq and protein analysis were performed for *n*=7 individuals per group (controls, alloimmunized patients, and non-alloimmunized patients). (A) Gating strategy for differential expression analysis. cTfh cells were identified as CXCR5^+^PD1^hi^ICOS^+^ cells. (B) Heatmap of the genes differentially expressed in cTfh cells for controls, alloimmunized patients and non-alloimmunized patients. The color scale indicates the mean fold-change in expression.
